# The effect of alcohol strength on alcohol consumption: findings from a randomised controlled cross-over pilot trial

**DOI:** 10.1186/s40814-021-00777-4

**Published:** 2021-01-30

**Authors:** Parvati R. Perman-Howe, Emma L. Davies, David R. Foxcroft

**Affiliations:** 1 King’s College London, Addiction Sciences Building, 4 Windsor Walk, Denmark Hill Campus, London, SE5 8BB UK; 2 Oxford Brookes University, Gipsy Lane Campus, Gipsy Lane, Oxford, OX3 0BP UK

**Keywords:** Alcohol, Alcohol strength, Public health, Prevention, Intervention, Licensed premises, Pub, Bar, Pilot trial, Feasibility trial

## Abstract

**Background:**

Reducing the alcohol content of drinks has the potential to reduce alcohol consumption. The aims of this study are to (1) test the feasibility of a randomised controlled trial (RCT) to assess the effect of alcohol strength on alcohol consumption within licensed premises in the United Kingdom (UK), and to (2) provide data to estimate key parameters for a RCT.

**Methods:**

This study is a double-blind randomised controlled cross-over pilot trial based within four licensed premises in the UK. Participants (*n* = 36) purchased and consumed ad libitum a 3.5% lager and a 4.8% lager during two separate study sessions. Descriptive statistics reported the efficacy and efficiency of the study processes, and the rates of licensed premises recruitment, and participant recruitment and attrition. Mean and the 95% confidence interval (CI) compared alcohol consumption between conditions. The mean, standard deviation (SD) and CI of UK units of alcohol consumed were used to calculate a sample size for a RCT. Responses to participant questionnaires and duration of participation in study sessions between conditions were analysed.

**Results:**

Components of the study protocol were effective and efficient. The venue recruitment rate was less than anticipated. The participant recruitment rate was greater than anticipated. The rate of attrition was 23% and varied by less than 1% according to the arm of the trial. There was a reduction of alcohol consumed under the intervention conditions. Estimated mean difference, and 95% CI (UK units): − 3.76 (− 5.01 to − 2.52). The sample size required for a RCT is 53. Participants did not find one lager more pleasant in taste: (on a scale of one to 10) − 0.95 (− 2.11 to 0.21). Participants found the reduced-strength lager less enjoyable: (on a scale of one to 10) − 1.44 (− 2.64 to − 0.24) and they perceived themselves to be less intoxicated after consuming it: (on a scale of one to 10) − 1.00 (− 1.61 to − 0.40).

**Conclusion:**

A RCT is feasible with minor alterations to the study protocol and scoping work to establish different brands of alcohol that are more alike and more enjoyable than the products used in the pilot trial.

**Trial registration:**

Registered in the American Economic Association (AEA) Randomised Controlled Trial (RCT) Registry as of 16 June 2017. Unique identifying number: AEARCTR-0002266.

**Supplementary Information:**

The online version contains supplementary material available at 10.1186/s40814-021-00777-4.

## Key messages regarding feasibility

- The uncertainties regarding feasibility were whether processes outlined in the study protocol were achievable, a sufficient number of licensed premises and participants could be recruited and retained, data support the hypothesis that people consume fewer UK units of alcohol when they consume reduced-strength lager and the sample size derived from pilot trial data is achievable for a definitive RCT.
- The key feasibility findings are: a RCT to assess the effect of alcohol strength on alcohol consumption within licensed premises in the UK is feasible.
- The implications of the feasibility findings for the design of the main study are: scoping work should be undertaken to establish brands of alcohol that are more alike and more enjoyable than those used in the pilot trial. Additionally, minor amendments to the protocol would improve the efficiency of a RCT. For example, recruiting licensed premises simultaneously rather than consecutively.

## Background

Excessive alcohol consumption is the leading cause of premature mortality, ill health and disability amongst those aged 15 to 49 in England [1]. Moreover, it is the fifth leading risk factor for ill health across all ages in England [1]. In 2018, there were 7551 avoidable deaths in the UK that were directly caused by alcohol [2]. A study with over 55,000 UK participants found that of the 69% who reported drinking alcohol, 27% reported drinking at levels that are classed as high risk [3]. Furthermore, 2.5 million people who regularly drink alcohol report exceeding weekly alcohol thresholds in a single drinking occasion [4]. In 2016, alcohol-related harm was estimated to cost UK society £47 billion [1, 5].

The most effective alcohol harm prevention interventions may be those that target sub-conscious processes, such as habits and cues, and that are readily scalable to the population level [6–10]. These include interventions that alter the properties of external stimuli, such as the strength of alcoholic drinks [9–11]. Such interventions could be especially beneficial in situations where people may not have direct access to important information. For instance, within licensed premises, lager taps display a brand logo but often do not display information about the strength of the product. Labelling drinks as lower in strength has been shown to increase the amount of alcohol consumed within a laboratory setting [12]. However, we propose that when information about alcohol strength is not forthcoming, such as when lager is purchased from the tap, most consumers will not consciously seek this information. Therefore, consumers cannot knowingly compensate for drinking lower-strength alcohol. Reducing the alcohol content of popular lager products that are sold on tap, or in other situations where information about alcohol content is not readily available, may lead to a reduction in alcohol consumption. Interventions that utilise sub-conscious processes have the added benefit of potentially reducing health inequalities as their recipients are not required to be health literate, numerate or have high-functioning cognition: lack of which are more prevalent with higher levels of deprivation [8, 13].

Reducing the alcohol content of drinks as a way to reduce alcohol consumption was proposed by the UK Coalition Government (2010 to 2015) as part of the Public Health Responsibility Deal (PHRD) [14]. Between 2011 and 2013, 1.3 billion UK units of alcohol were removed from the UK market by reductions in the alcohol content of drinks. However, this only equated to the average strength of beer falling by 0.28% alcohol by volume (ABV) [15]. In 2016, the world’s largest brewer, Anheuser-Busch InBev (AB InBev) launched their “Global Smart Drinking Goals” campaign [16]. One of their goals was to “ensure no- or lower- (≤ 3.5% ABV) alcohol products represent at least 20% of AB InBev’s global beer volume by the end of 2025” [16]. Although initially this appears promising from a public health perspective, there are concerns that AB InBev will expand their portfolio by creating new brands of no- and lower-alcohol beer rather than reformulating their current products to contain less alcohol. Inevitably, these new brands will be heavily marketed, and research shows that marketing tactics used for reduced-strength wine and beer can lead to an increase in alcohol consumption [12, 17]. Data from a Norwegian study found that when availability of lower-strength drinks increased, people were more likely to consume it as an addition to, rather than a replacement for, stronger alcoholic drinks [18]. Therefore, it is unlikely that adding new reduced-strength brands to the market will decrease average alcohol consumption and, instead, may have an opposite and detrimental effect. The most effective mechanism that may explain how reducing the alcohol content of drinks could reduce alcohol consumption is by current drinkers replacing the alcoholic drinks they normally consume with lower-strength alternatives and without increasing the volume of alcoholic drinks consumed [19].

There is a paucity of evidence to support initiatives to reduce the strength of alcoholic drinks. Most studies of alcohol strength are strength discrimination studies. The majority of these were laboratory-based [20–24] and one study was based within a mocked-up lounge in a community centre [25]. All but one incorporated beer, or beer and spirits, and a single study focused on wine [23]. These studies all support the hypothesis that people cannot readily distinguish between alcoholic drinks of different strength, which indicates that there is potential to subconsciously alter alcohol consumption by altering the ABV of alcoholic drinks. An experiment with Canadian students found that participants could not discriminate between beers of 3.8% ABV and 5.3% ABV and, importantly, similar levels of enjoyment and perceived intoxication were reported between conditions [26]. However, this study had numerous limitations: it used a small sample of male students, it was based within a classroom and participants were restricted to the amount of alcohol they could consume. A more robust study that assessed the effect of the strength of beer and mixed spirit-based drinks on consumption supports the hypothesis that reducing the alcohol content of drinks does not lead to an increase in the volume of alcohol consumed, therefore reducing consumption [27]. These findings contradict the titration hypothesis, which is commonly used as a counter argument for reducing the alcohol content of drinks. The titration hypothesis states that individuals will adjust their intake of a substance to reach a desired level of intoxication [28]. Although, to date, this is the only robust experimental study to assess the effect of alcohol strength on alcohol consumption within a naturalistic setting, there are limitations in its design. Most notably it was based within closed student fraternity parties comprising a single fraternity at one university in the United States of America (USA) [27].

High-quality research is warranted to assess the effect of alcohol strength on consumption within a naturalistic environment. Prior to a definitive RCT, a pilot study was required to test feasibility and estimate key parameters for the RCT’s design. This study aimed to pilot a double-blind randomised controlled cross-over trial to assess the effect of alcohol strength on alcohol consumption in a single drinking occasion within licensed premises in the UK.

### Objectives

The objectives were to establish whether:

- components of the study protocol were efficient and worked together, or could be amended to be or do so
- licensed premises recruitment rate was at least one per month for a minimum of 4 months or until four licensed premises were recruited
- participant recruitment rate was at least four per initial study session for each cohort
- participant attrition was 30% or less and this did not vary by more than 10% according to the arm of the trial
- estimations of the mean and 95% CI of the number of UK units of alcohol consumed by participants in a single drinking occasion support the hypothesis that people consume fewer UK units of alcohol when they consume reduced-strength lager
- the sample size calculated from data obtained in the study is achievable for a definitive RCT.

## Methods

### Trial design

A double-blind randomised controlled AB/BA cross-over pilot trial was implemented. The study was defined as a randomised pilot trial in accordance with Eldridge et al.’s conceptual framework for defining feasibility and pilot studies in preparation for a RCT [29]. That is, the future RCT, or parts of it, including the randomisation of participants, were conducted on a smaller scale to see if it could be done. Additionally, and in line with Teare et al.’s definition of a pilot study, it provided data with which to estimate key parameters for the design of a RCT [30].

#### Changes to methods

The study was designed to include a 4-week washout period between each participants’ two study sessions. However, due to participants’ availability, the minimum washout period was reduced to 2 weeks. This was deemed adequate for participants to have desensitised to the sensory aspects of the alcohol they consumed in their first study session. Additionally, there was no risk of carryover effects from the alcohol consumed during participants’ first study session as alcohol is expelled from the body at the rate of approximately one unit per hour.

### Participants

Participants were required to meet all of the inclusion criteria and not meet any of the exclusion criteria (Table 1).

**Table 1:** Eligibility criteria

Inclusion	Exclusion
18 years of age or older	Has ever sought help, or been treated, for an alcohol dependency
Regular drinker of lager within a licensed premises (≥ once in the past three months)	Has an illness or condition with which they should not be consuming alcohol
Able to attend two study sessions	Is on medication with which they should not be consuming alcohol
Provides informed consent	Pregnant
	Has a breath alcohol concentration (BrAC) > 35 μg/100 ml breath when they arrive for a study session

#### Settings

Four licensed premises in the South East of England each hosted four study sessions (Table 2). Multiple sites were used in order to increase the chances of fulfilling the sample and to enhance its representativeness [31].

**Table 2:** Participating licensed premises

	Type of licensed premises	Dates of study sessions	Study session details
Venue one	Cricket club bar	May and June 2018	Fridays (fortnightly) during bar, BBQ and children’s coaching event 18:00 until closing
Venue two	Village pub	August to October 2018	Thursdays (fortnightly) 18:00 until closing
Venue three	Students’ Union bar	October and November 2018	Sundays (fortnightly) during quiz event 20:00 until closing
Venue four	Students’ Union bar	February and March 2019	Tuesdays (fortnightly) during quiz and karaoke event 20:00 until closing

#### Venue recruitment

**Fig. 1:**
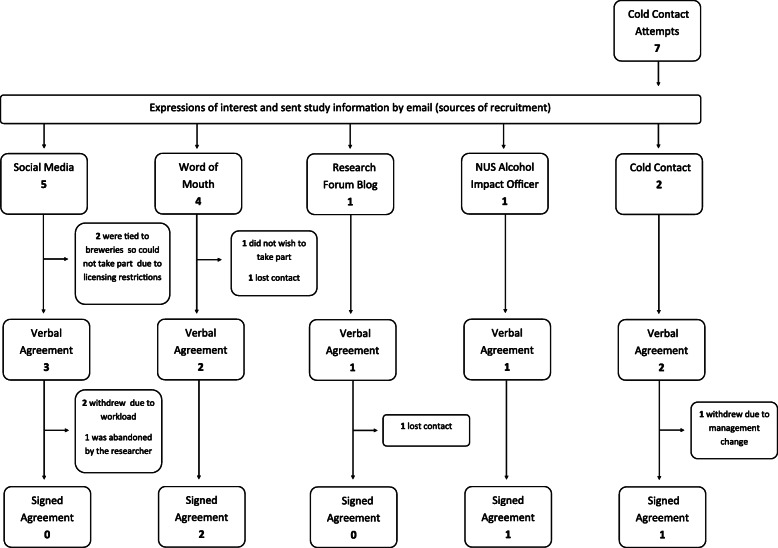
Licensed premises recruitment pathways

The four licensed premises were recruited in an iterative process (Fig. 1). Licensed premises were approached in an ad hoc manner based on the principal investigator (PI)’s contacts/knowledge of local licensed premises. Landlords/managers were incentivised to take part with the offer of £500 for hosting four study sessions.

#### Participant recruitment

The PI asked participating licensed premises managers to display posters, and hand out flyers, within their venue and to post recruitment advertisements on their social media accounts. These guided people to contact the PI via email or telephone for further information about the study. The PI sent those who subsequently contacted them an invitation letter and a participant information sheet (PIS), which both included a link to an online eligibility survey that people were asked to complete if they wanted to take part in the study. The PI analysed responses to the eligibility survey and emailed those who were eligible to confirm dates for their two study sessions. Study materials are published on the open science framework (OSF) website: https://osf.io/htx2b/.

Following feedback from the manager of venue one, one face-to-face recruitment session was conducted at each participating licensed premises, alongside the initial methods of recruitment, to increase the recruitment rate. During recruitment sessions study information was provided, people could complete the eligibility survey and those who were eligible could confirm the dates for their study sessions.

Written informed consent was taken when participants arrived at the licensed premises for their first study session.

Participants were incentivised to take part with the offer of being entered into a free prize draw to win one of two prizes of £100.

### Intervention/control

#### Intervention product

The intervention product was Bud Light (BL) lager: 3.5% ABV. Cans (440 ml) of BL were wrapped in duct tape to conceal their branding and chilled in a fridge 24 h before a study session. Before each study session, the cans were transferred to a fridge within the hosting venue. The lager was poured from the 440 ml cans into a pint glass so that a full pint (568 ml) was served: each pint therefore contained more than one can of lager. Each pint was sold for approximately 2/3s of the price of the cheapest pint of lager normally sold at the venue. Participants could purchase and consume the intervention product ad libitum during their study session.

#### Control product

The taste-matched control product was Becks (B) lager: 4.8% ABV. It was prepared, sold and served identically to the intervention product. The pre-published protocol explaining how the intervention and control products were taste matched can be accessed on the OSF website: https://osf.io/zndrj/.

### Outcomes

#### Feasibility criteria

The primary outcome was the feasibility of a RCT. A RCT was deemed feasible if it met the following pre-specified criteria [32]:

1. Components of the study protocol were efficient and worked together or could be amended to be or do so. These included:
  the administration of data collection tools
  the consent process
  the randomisation process
  data management processes
  the roles and requirements of study personnel
2. The licensed premises recruitment rate was at least one per month for a minimum of 4 months or until four licensed premises had been recruited.
3. The participant recruitment rate was at least four per initial study session for each cohort.
4. The rate of attrition was 30% or less and this did not vary by more than 10% according to the arm of the trial.
5. Estimations of the mean and 95% CI of the number of UK units of alcohol consumed by participants in a single drinking occasion, when they consume BL, and B, suggest that people consume fewer UK units of alcohol when they consume reduced-strength lager.
6. The sample size for a RCT, calculated from data obtained in this pilot trial, is achievable based on the recruitment rates of licensed premises and participants and the rate of participant attrition.

#### Outcome measurements

The PI kept records that covered point 1 of the feasibility criteria (see the“Feasibility criteria” section). Feedback from members of the research team, participating licensed premises, and participants were obtained and recorded throughout the study.

Electronic datasets were used to record:

- licensed premises that were approached
- landlords/managers who expressed willingness to participate
- landlords/managers who signed a letter of access
- participants who consented to participate (and at each separate participating licensed premises)
- participants who consented and did not complete two study sessions
- participants who consented and dropped out during or after the intervention study session
- participants who consented and dropped out during or after the control study session

To measure the number of study-specific drinks served, the research assistant (RA) stamped participants’ randomisation cards each time they purchased a study-specific drink. Participants were asked to return their pint glass to the RA if they did not finish all of a study-specific drink (with the remainder of the drink left in the glass). The PI measured the amount of alcohol that had been left in the glass and converted this to UK units of alcohol. The randomisation cards were returned to the PI at the end of each study session. The PI then quantified the number of UK units of alcohol served to each participant (as indicated on the randomisation card) and deducted the number of UK units of alcohol that each participant had left in their glass from this total. This provided a measure for the amount of alcohol each participant consumed during a study session. The number of UK units of alcohol were also converted to, and displayed as, grams of alcohol for an international audience.

After venue one had completed their four study sessions, an extra measure was put in place: the duration of participation (of each individual) in each study session. This was added as an additional indicator of whether participants’ drinking behaviour differed between conditions after it was observed that some participants had signed out from their study session but remained within the venue.

### Sample size

As there were no data from previous studies on which to base a statistical calculation and there is no consensus in the literature about the required sample size for pilot trials, the sample size was calculated using preliminary datasets. These preliminary datasets were based on the hypothesis that there is no significant difference between the number of alcoholic drinks individuals consume regardless of their ABV, which has been shown in a previous study [27]. The sample size was calculated using the R statistical package “pwr” [33, 34]. The level of statistical significance was set at 5% and power at 80%. The sample size for a two-sided paired *t* test was calculated as 52: 52 participants participating in two trial arms (see Additional file 1 for more details). As this did not account for attrition, participants who dropped out of the pilot trial were replaced. However, due to time constraints, we did not achieve our target sample size.

#### Trial withdrawal

Participants who wished to withdraw from the study were directed to contact the PI.

Participants who were seen, by the PI, the RA or other members of staff at the hosting venue, to be obviously and persistently breaching the protocol were withdrawn from the study.

### Randomisation

#### Sequence generation

Participants were randomly assigned to the order that they received the intervention (BL) and the control (B), using the AB/BA format to counterbalance conditions. A separate computer generated randomisation sequence was produced for each study venue using Randomization.com software [35].

### Concealment

The first “treatment” label (pink or purple) designated to each subject in the randomisation sequence was translated as a discrete, coloured label on a randomisation card that was concealed in a sealed and numbered opaque envelope. The sealed envelopes were placed in a pile, which was overturned and secured once all envelopes were present so that the sequence was in ascending numerical order.

### Implementation

The chief investigator (ChI: DF) generated the allocation sequence and concealed the allocation. The PI enrolled participants and assigned them to their sequence (AB/BA) by asking them to take the next numbered envelope from the pile and opening it.

### Blinding

The participants and the RA were blinded to the intervention and control products and the order in which they were assigned.

Randomisation cards displayed a colour-coded label, and the participants and the RA were unaware of the colour-coding system. Coloured labels were placed on the de-identified lager cans, which corresponded to the coloured labels on the randomisation cards. The RA asked the participants to show their randomisation cards when they purchased a study-specific drink and the colour of the label on the card informed the RA which drink to serve.

### Statistical methods

The efficacy and efficiency of the study processes, and the rates of licensed premises recruitment, and participant recruitment and attrition were analysed and reported using descriptive statistics.

Mean value and 95% confidence intervals (CIs) were used to compare the number of UK units of alcohol consumed, the mean duration of participation in study sessions and responses to participant questionnaires, between the study conditions.

The mean, SD and CI of the number of UK units of alcohol consumed were used to calculate a sample size for a definitive RCT. The sample size was based on the smallest effect size in the CI for the mean difference in alcohol consumption between study conditions.

## Results

### Licensed premises recruitment

Licensed premises were recruited between October 2017 and December 2018.

### Participant recruitment

Participants were recruited between April 2018 and February 2019.

#### End of study

The study ended, as planned, after four licensed premises had completed four study sessions each. The study officially ended when the final participant was sent a debrief email/letter.

### Baseline characteristics

**Fig. 2:**
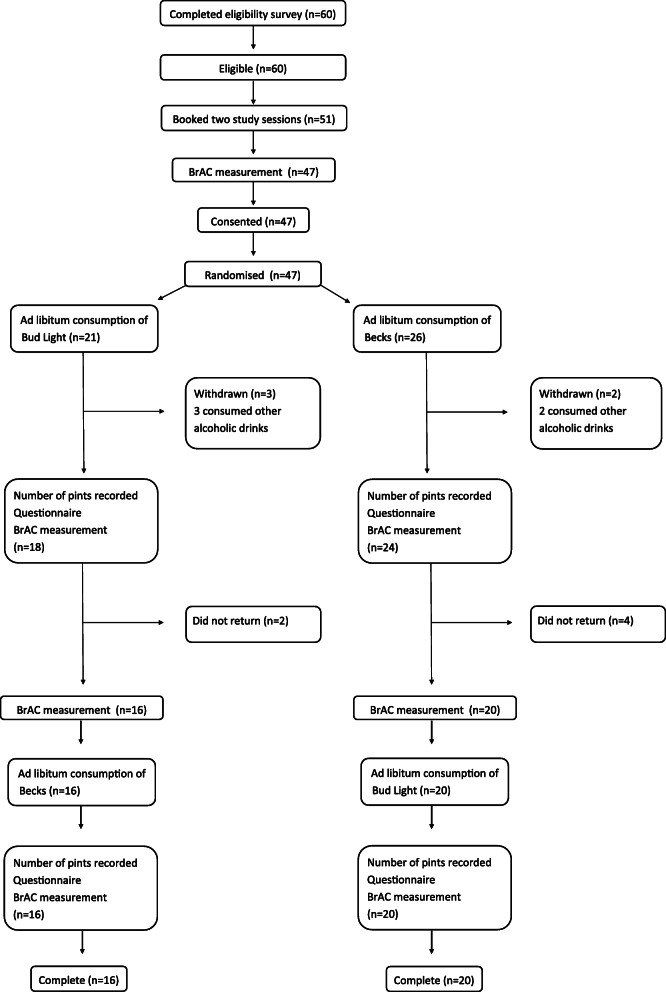
Participant pathways

Thirty-six participants completed the pilot trial (Fig. 2). Participant characteristics can be seen in Table 3.

**Table 3:** Participant baseline characteristics

Mean age (years)	30.7 (SD = 13.59), range = 18 to 66
Gender	89% M, 11% F
Employment status	42% students, 36% worked full-time, 11% self-employed, 3% worked part time, 3% retired, 3% unemployed seeking work, 3% unemployed not seeking work
Participants from each venue	28% from each of venues one, two and three, 17% from venue four

### Numbers analysed

Data from all 36 participants were included in the estimation of the mean and 95% CI of the number of UK units of alcohol consumed by participants when they drank BL, and B. Data from 26 participants were included in the analysis of the duration of participation in study sessions as this measure was added once data collection had started.

### Outcomes and estimation

According to the study protocol, a RCT would be deemed feasible if the pilot study met six criteria for success [32].

1. Components of the study protocol were efficient and worked together or could be amended to be or do so. These include:

- The administration of data collection tools

The administration of data collection tools was adequate. Some participants found the breathalyser overly sensitive and it took them up to 5 min to provide a measurement. Additionally, some participants struggled to interpret question six on the eligibility survey: “Roughly how many drinks did you have on your heaviest drinking occasion in the last year?”. These incidents, however, had minimal impact on the BrAC measurements, which were not subsequently analysed, and the outcome of the eligibility survey. The eligibility survey, the questionnaires and the storage of data on the randomisation cards and the schedule spreadsheet were all suitable means of capturing data and only minor amendments are required to increase their efficiency.

- The consent process

The consent process was simple and efficient and was applied without incident.

- The randomisation process

The randomisation process was efficient, although there was one sequence error whereby a participant took the second randomisation envelope from the pile rather than the next envelope. This occurred when the PI was busy with multiple participants and the incident could have been prevented if the RA’s role was expanded to increase the research team’s capacity.

- Data management processes

Data management processes were simple to follow, and they were effective at ensuring the data were both secure but accessible to those who required it.

- The roles and requirements of study personnel

Three study personnel were required to enact the pilot trial: the PI, the RA and the ChI. The PI undertook all roles aside from those that the RA and ChI were required to undertake to ensure the trial was a double blind. The RA had limited responsibilities: pouring the study-specific drinks, exchanging them for a cash payment and notifying the PI when participants reached their consumption threshold. There was scope for the RA’s role to be expanded to reduce the congestion that occurred when multiple participants signed into the study simultaneously. The ChI was required to prepare the randomisation sequences and the randomisation cards and to ensure concealment. The PI provided them with instructions, and feedback from the ChI suggested that the process was straightforward, reproducible and efficient.

2. The licenced premise recruitment rate was at least one per month for a minimum of 4 months or until four licenced premises were recruited.

In total, four licensed premises were recruited in an iterative process (Fig. 1). Venue recruitment ran for 14 months and 3 days between October 2017 and December 2018. The recruitment rate was one venue every 107 days: approximately one venue every three and a half months.

3. Participant recruitment rate was at least four per initial study session for each cohort.

Sixty people completed the eligibility survey and provided contact details: 44 (73%) were completed in paper format during a recruitment or study session and 16 (27%) were completed electronically: five of the latter were completed on the PI’s laptop during a study session at venue four because there was a shortage of paper forms. One hundred percent of those who completed the eligibility survey were eligible and 51 (85%) booked study sessions. Forty-seven people (92% of those who booked study sessions) consented and all of these were randomised. The participant recruitment rate was 5.9 per initial study session for each cohort.

4. The rate of attrition for the pilot trial was 30% or less and this did not vary by more than 10% according to the arm of the trial.

Thirty six of 47 participants completed the pilot trial (77%). The rate of attrition was 23%. The rate of attrition varied by less than 1% according to the order in which participants were randomised to the intervention and the control conditions: 24% in the BL-B arm and 23% in the B-BL arm.

5. Estimations of the mean and 95% CI of the number of UK units of alcohol consumed by participants in a single drinking occasion, when they consume BL and B, suggest that people consume fewer UK units of alcohol when they consume reduced-strength lager.

There was a notable reduction in alcohol consumption when participants consumed the reduced-strength lager (the intervention condition). The estimated mean difference in alcohol consumed by participants when they consumed the reduced-strength lager, BL, compared to the regular-strength lager, B, was − 3.76 UK units SD = 3.69 (− 5.01 to − 2.52) or − 30.56 grams (g) SD = 29.83 (− 40.65 to − 20.46) (Table 4; see Additional file 2 for data by recruitment site/gender/student vs non-student). Data illustrate that participants consumed 31% less alcohol when they consumed ad libitum a 3.5% ABV lager compared to a 4.8% ABV lager.

**Table 4:** Pilot trial data

	Mean (reduced-strength lager, *n* = 36), SD, (95% CI)	Mean (regular-strength lager, *n* = 36), SD, (95% CI)	Mean difference (mean reduced-strength lager minus mean regular-strength lager), SD, (95% CI)
Alcohol consumption (UK units)	8.28, SD = 4.17 (6.87 to 9.69)	12.04, SD = 5.33 (10.24 to 13.84)	− 3.76, SD = 3.69 (− 5.01 to − 2.52)
Alcohol consumption (grams)	65.78, SD = 33.51 (54.44 to 77.12)	96.34, SD = 42.61 (81.92 to 110.75)	− 30.56, SD = 29.83 (− 40.65 to − 20.46)
Pints consumed	4.14, SD = 2.09 (3.43 to 4.84)	4.45, SD = 1.96 (3.79 to 5.12)	− 0.31, SD = 1.51 (− 0.82 to 0.20)
Study session duration (hh:mm)	2:33, SD = 0:51 (2:12 to 2:53)	2:39, SD = 0:52 (2:18 to 3:00)	− 0:06, SD = 0:41 (− 0:23 to 0:10)
Pleasantness of taste	4.86, SD = 2.73 (3.94 to 5.79)	5.81, SD = 2.13 (5.09 to 6.53)	− 0.95, SD = 3.43 (− 2.11 to 0.21)
Enjoyment	4.79, SD = 2.79 (3.53 to 5.89)	6.23, SD = 2.21 (5.40 to 7.27)	− 1.44, SD = 3.54 (− 2.64 to − 0.24)
Perceived intoxication	4.09, SD = 1.91 (3.44 to 4.73)	5.09, SD = 1.97 (4.42 to 5.76)	− 1.00, SD = 1.79 (− 1.61 to -0.40)

6. The sample size for a RCT, calculated from data obtained in this pilot trial, is achievable based on the recruitment rates of licensed premises and participants and the rate of participant attrition.

A conservative sample size was estimated based on the lowest effect size in the 95% CI for the mean difference in the number of UK units of alcohol consumed between study conditions (− 2.52). Taking account of the within subject design (correlation between repeated measures), Cohen’s *d* was calculated as 1.01 (0.67 to 1.35) [36]. This gave an estimated sample size for a future trial of *n* = 43 [34]. This is based on the lowest effect size (0.67), power of 0.95, and a type I error rate of 0.01. Given anticipated attrition of 23%, the suggested target sample size to be recruited for a definitive RCT is 53.

An average of nine participants completed, and two participants did not complete, the trial at each study venue that hosted four study sessions. It is therefore estimated that six venues would be required to host four study sessions each during a definitive RCT with a sample size of 53. Based on the recruitment rate of the pilot trial, six licensed premises could be recruited in approximately 21 months. It is expected that each of these venues would have completed their four study sessions within 3 months of being recruited. This means that it would take approximately 24 months to complete a RCT with 53 participants. However, this should be regarded as a worst-case scenario where venue recruitment is consecutive rather than simultaneous. If venues were to be recruited simultaneously, it would take significantly less time to complete a definitive RCT.

### Ancillary analyses

To assess whether the witnessed trend of a reduction in alcohol consumed under the intervention condition could be due to factors other than the strength of the lager, further analyses were undertaken (Table 4; see Additional file 2 for data by recruitment site/gender/student vs non-student).

Data show that:

- No difference was detected in the number of pints participants consumed between study conditions. The estimated mean difference in the number of pints consumed (BL compared to B) was − 0.31 SD = 1.51 (− 0.82 to 0.20)
- No difference was detected in the duration of participation in study sessions based on whether participants were consuming BL or B: estimated mean difference in study session duration (BL compared to B) was − 0:06 SD = 0:41 (− 0:23 to 0:10)
- Participants did not find one lager product more pleasant in taste than the other. The estimated mean difference of the reported pleasantness of taste (BL compared to B) on a scale of one to 10 was − 0.95 SD = 3.43 (− 2.11 to 0.21)
- Participants rated B as being more enjoyable than BL. The estimated mean difference of reported enjoyment (BL compared to B) on a scale of one to 10 was − 1.44 SD = 3.54 (− 2.64 to − 0.24)
- Participants perceived themselves to be more intoxicated at the end of the study session in which they had been consuming B compared to BL. The estimated mean difference of reported levels of intoxication (BL compared to B) on a scale of one to 10 was − 1.00 SD = 1.79 (− 1.61 to − 0.40).

**Fig. 3:**
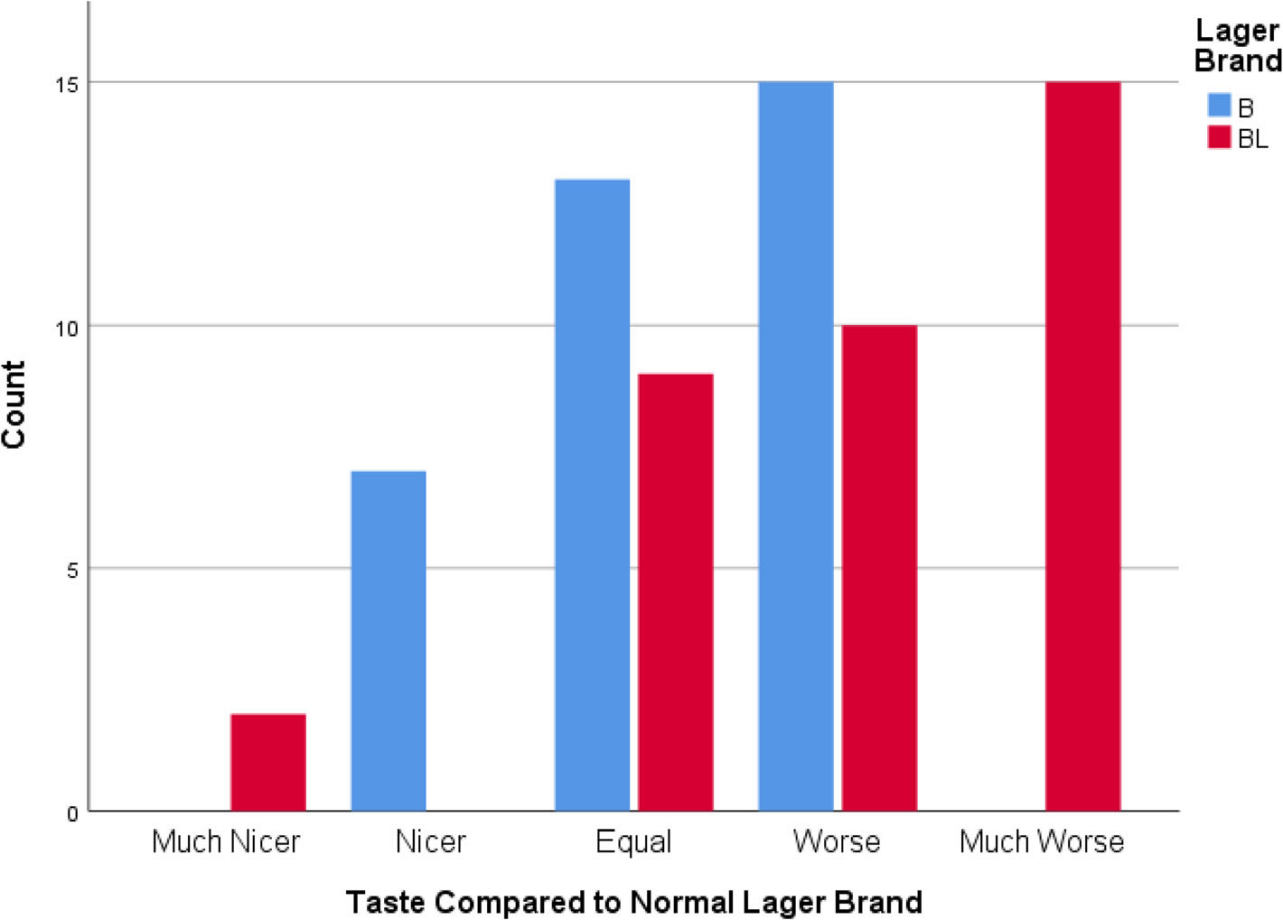
Participants’ ratings for the taste of each study-specific lager compared to the lager brand they normally consume

When participants compared the taste of the study-specific lager with their regular brand of lager, participants were more likely to give a negative response (much worse than my normal drink or worse than my normal drink) than a positive or neutral response for both BL (25/36) and B (15/35). The mode for BL was the response “much worse than my normal drink”, whilst the mode for B was “worse than my normal drink” (Fig. 3).

### Harms

No adverse events were reported to the PI.

## Discussion

This pilot trial uncovered some issues with the study protocol, including inadequately matched control and intervention products, which should be addressed prior to a definitive trial. Whilst the venue recruitment rate was less than anticipated, the participant recruitment rate was greater than anticipated. The rate of participant attrition was 23% and varied by less than 1% according to the arm of the trial. There was a 31% reduction in alcohol consumed when participants consumed the reduced-strength lager. Based on data from this pilot trial, the sample size required for a RCT is 53.

### Limitations

One of the limitations of the pilot trial is the uncertainty as to whether participants adhered to the study protocol and only consumed the study-specific lager that was recorded on their randomisation card. There is also the possibility that participants bought study-specific drinks for non-participants as they were cheaper than the regular lager sold at each venue. Either of these situations would have resulted in inaccurate data on alcohol consumption. To reduce the risk of collecting inaccurate data, researchers could have been placed within the venues during study sessions to covertly observe participants’ drinking behaviour and remove deviant participants from the study. A similar strategy was successfully implemented in a study assessing the effect of serving size on alcohol consumption within licensed premises in the UK [37]. In this study, six researchers posed as patrons within participating licensed premises during each study session to covertly observe participants’ alcohol consumption. In the current study, the PI and the RA did observe participants’ drinking behaviour throughout the pilot trial study sessions; however, there were not enough resources to officially observe participants. This should be considered for future iterations of the trial.

Another limitation can be inferred from the questionnaire findings: the intervention and control products were sub-optimally matched. If the pilot trial participants were aware that they were consuming different strength products, then this may have biased their drinking behaviour and questionnaire responses due to lack of blinding. Furthermore, when participants were asked to rate BL, and B in comparison to their regular brand of lager, the most popular responses were “much worse” or “worse”. This indicates that neither drink tasted favourable to the participants, and BL was less favourable than B. Prior to a future trial, further exploratory work should be undertaken to establish an intervention and a control product with improved matching characteristics, and that evoke equal levels of enjoyment.

Furthermore, the term “efficient” in feasibility criterion 1 was not pre-defined. Therefore, the decision as to whether components of the study protocol worked efficiently was subjective.

Another limitation is that the study findings do not translate to other settings. For example, pilot trial data suggest that a definitive RCT to assess the effect of alcohol strength on alcohol consumption is feasible to enact within licensed premises in the UK. However, they do not tell us the feasibility of undertaking the study within the home setting, or within licensed premises in different countries. When designing a study to assess the effect of alcohol strength on alcohol consumption in a different setting, lessons could be learnt from the findings of this pilot trial. However, it is likely that significant amendments to the study protocol would have to be made. For example, a study in the home setting would require different methods for administering the study processes (such as consent and randomisation) and data collection tools; supplying, and regulating the supply of, study-specific alcohol; and monitoring and recording alcohol consumption. Therefore, such a trial would need to be piloted before it is implemented as a definitive RCT.

### Interpretation

There are four possible outcomes of a pilot study [38]:

1. *Stop*: a main study is not feasible.
2. *Continue*, *but modify protocol*: a main study is feasible but requires modifications.
3. *Continue without modifications but monitor closely*: a main study is feasible but requires close monitoring.
4. *Continue without modifications*: a main study is feasible without modifications.

Option two most accurately describes the outcome of this pilot trial: A RCT is feasible with better matched intervention and control products and minor protocol amendments.

A definitive RCT should be very similar to the pilot trial but with minor alterations to the study processes based on data from the pilot trial. Prior to a RCT, scoping work is required to establish whether different brands of beer/lager would be more favourable options for the intervention and control products. Scoping work should also aim to establish control and intervention products that are more accurately matched in a broader range of aspects including carbonation, colour, smell and enjoyability. This scenario would require co-production between the researchers and the public to help shape and guide the research. It would therefore be more resource intensive than the pilot trial, but it would likely increase the validity of the findings.

To conclude, this pilot trial has demonstrated that a RCT to assess the effect of alcohol strength on alcohol consumption within licensed premises in the UK is feasible with better matched intervention and control products and minor protocol amendments.

The protocol for the pilot trial has been published as an open access journal article [32].

## Supplementary Information

**Additional file 1.** Preliminary datasets for the sample size calculation. An explanation of how the pilot trial sample size was calculated including the preliminary datasets used

**Additional file 2.** Pilot trial data by recruitment site/gender/student vs non-student. Table of pilot trial data (mean, SD, 95% CIs) by recruitment site/gender/student vs non-student

## Data Availability

The datasets generated and/or analysed during the current study are available in the OSF repository [40].

## Abbreviations

ABV: Alcohol by volume
AB InBev: Anheuser-Busch InBev
B: Becks lager
BL: Bud Light lager
BrAC: Breath alcohol concentration
ChI: Chief investigator
CI: Confidence interval
CIs: Confidence intervals
OBU: Oxford Brookes University
OSF: Open science framework
PHRD: Public Health Responsibility Deal
PI: Principal investigator
PIS: Participant information sheet
RA: Research assistant
RCT: Randomised controlled trial
SD: Standard deviation
UK: United Kingdom
UREC: University Research Ethics Committee
USA: United State of America
