# Supplementary material for: The effect of alcohol strength on alcohol consumption: findings from a randomised controlled cross-over pilot trial
Source: Pilot Feasibility Stud. 2021 Jan 30;7:37. doi: 10.1186/s40814-021-00777-4 (PMC7846989; doi:10.1186/s40814-021-00777-4)
Supplement: Supplementary file 1 — Additional file 1. Preliminary datasets for the sample size calculation. An explanation of how the pilot trial sample size was calculated including the preliminary datasets used [file 40814_2021_777_MOESM1_ESM.docx]

Preliminary datasets for the sample size calculation

The sample size calculation for the pilot trial was not undertaken to enable formal hypothesis testing but to provide the most accurate estimate of the effect size and variance necessary to plan a RCT. The sample size was calculated using preliminary datasets as there were no data from previous studies on which to base a statistical calculation.

Firstly, data for the estimated age of patrons from a range of licensed premises that were similar to those being targeted for study recruitment were obtained by corresponding with premises managers. The managers also provided an estimate of the proportion of male to female patrons. These estimates accounted for all adult patrons within the licensed premises regardless of the type of product they consume: it was deemed too complex to gage age and gender estimates for lager drinkers only.

Once the consumer profiles had been obtained, preliminary datasets for 40 participants were created; 40 was the estimated average attendance across the venues at any one time. The principal investigator (PI) inputted 40 representative individual ages into an online calculator from which the mean age and the standard deviation (SD) were calculated. These data informed the calculation of mean consumption of UK units of alcohol at each venue. Data from the Office for National Statistics^[[1]](#endnote-1)^ displayed the proportion of individuals within different age brackets who consumed either 4.67 units or less, 4.68 to seven units, seven to 14 units or more than 14 units on their heaviest drinking occasion within one week. All the venues except the student bar had an estimated average consumer age within the 25 to 44 age bracket. The student bar had an estimated average consumer age within the 16 to 24 age bracket. Table 1 displays alcohol consumption (on the heaviest day of drinking in a week) in the UK within each of these two age brackets.

*Table 1:* *Alcohol consumption on heaviest day of drinking in a week for different age brackets*

|  | **Number of UK units of alcohol consumed** | | | | |
| --- | --- | --- | --- | --- | --- |
| ***Age bracket*** | **≤4.67** | **4.68 to 7** | **>7 to 14** | **>14** | **Total** |
| **16 to 24** | 41% | 16% | 26% | 17% | 100% |
| **25 to 44** | 47% | 14% | 28% | 11% | 100% |

These data were converted to frequencies for each hypothetical study venue based on the average age of their consumers (Table 2).

*Table 2: Estimated alcohol consumption on heaviest day of drinking in a week based on the average age of consumers within each hypothetical study venue*

|  | **Number of UK units of alcohol consumed** | | | | |
| --- | --- | --- | --- | --- | --- |
| **Venue** | **≤4.67** | **4.68 to 7** | **>7 to 14** | **>14** | **Total consumers** |
| **Village cricket club bar** | 19 | 6 | 11 | 4 | 40 |
| **City gastro pub** | 19 | 6 | 11 | 4 | 40 |
| **City centre pub** | 19 | 6 | 11 | 4 | 40 |
| **Village pub with B&B** | 19 | 6 | 11 | 4 | 40 |
| **Student bar** | 16 | 7 | 10 | 7 | 40 |

Where the venue had a higher proportion of male to female consumers, this was accounted for in the calculations and the mean consumption increased accordingly: 52% of males drink more than 4.67 units on their heaviest drinking day compared to 37% of females; 12% of males drink over 14 units on their heaviest drinking day compared with 4% of women^1^ .

These data were used to model 40 participants’ normal alcohol consumption, and expected alcohol consumption with the intervention, at different hypothetical venues (see Table 3 for an example preliminary dataset for a student bar). Estimated mean number of UK units of alcohol consumed and SDs under normal conditions were calculated. The mean value for alcohol consumption was reduced by 27% to give the estimated average alcohol consumption in UK units under the intervention conditions. From these data the predicted mean difference was calculated by subtracting the mean value for the predicted number of alcohol units consumed under the intervention conditions from the mean value for the predicted number of alcohol units consumed under the control conditions.

| **Participant number** | **Normal consumption (UK units)** | **Expected consumption with intervention (UK units)** | **Difference in consumption (UK units)** |
| --- | --- | --- | --- |
| 1 | 1 | .75 | .25 |
| 2 | 1.6 | 1.2 | .4 |
| 3 | 1.6 | 1.2 | .4 |
| 4 | 2 | 1.4 | .6 |
| 5 | 2 | 1.4 | .6 |
| 6 | 2.4 | 1.6 | .8 |
| 7 | 2.4 | 1.6 | .8 |
| 8 | 3.2 | 2.3 | .9 |
| 9 | 3.2 | 2.3 | .9 |
| 10 | 4 | 3 | 1 |
| 11 | 4 | 3 | 1 |
| 12 | 4 | 3 | 1 |
| 13 | 4 | 3 | 1 |
| 14 | 4.4 | 3.1 | 1.3 |
| 15 | 4.4 | 3.1 | 1.3 |
| 16 | 4.4 | 3.1 | 1.3 |
| 17 | 4.8 | 3.4 | 1.4 |
| 18 | 5 | 3.6 | 1.4 |
| 19 | 5 | 3.6 | 1.4 |
| 20 | 6 | 4.5 | 1.5 |
| 21 | 6 | 4.5 | 1.5 |
| 22 | 6.6 | 4.6 | 2 |
| 23 | 7 | 4.9 | 2.1 |
| 24 | 8 | 5.7 | 2.3 |
| 25 | 8 | 5.7 | 2.3 |
| 26 | 8 | 5.7 | 2.3 |
| 27 | 10 | 7.4 | 2.6 |
| 28 | 10 | 7.4 | 2.6 |
| 29 | 10 | 7.4 | 2.6 |
| 30 | 10 | 7.4 | 2.6 |
| 31 | 12 | 8.6 | 3.4 |
| 32 | 12 | 8.6 | 3.4 |
| 33 | 14 | 10.2 | 3.8 |
| 34 | 15 | 11 | 4 |
| 35 | 15 | 11 | 4 |
| 36 | 16 | 11.8 | 4.2 |
| 37 | 16 | 11.8 | 4.2 |
| 38 | 16 | 11.8 | 4.2 |
| 39 | 18 | 13.6 | 4.4 |
| 40 | 20 | 14.8 | 5.2 |
| **Mean** | **7.68** | **5.60** | **2.08** |
| **SD** | **5.23** | **3.88** | **1.36** |

In the above example (Table 3), the mean difference was estimated as -2.08 UK units of alcohol. For the sample size calculation, the PI used a conservative estimate for the SD in the intervention arm: the same SD as in the control arm (5.23). Table 3 summaries the preliminary datasets for all five hypothetical study venues.

*Table 3: Summary of preliminary datasets created for five hypothetical study venues*

| **Venue** | **Mean consumer age (±SD)** | **Proportion of male consumers (%)** | **Mean consumption in units under normal conditions (±SD)** | **Mean consumption in units with intervention (±SD)** | **Difference in mean consumption in units** |
| --- | --- | --- | --- | --- | --- |
| **Village cricket club bar** | 37.58 (±10.92) | 60 | 6.64 (±4.31) | 4.85 (±4.31) | -1.79 |
| **Gastro pub** | 36.63 (±13.59) | 50 | 6.52 (±4.27) | 4.76 (±4.27) | -1.76 |
| **City centre pub** | 34 (±13.20) | 50 | 6.52 (±4.27) | 4.76 (±4.27) | -1.76 |
| **Student bar** | 19.88 (±1.89) | 50 | 7.68 (±5.23) | 5.60 (±5.23) | -2.08 |
| **Village pub with B&B** | 36.88 (±16.06) | 70 | 6.79 (±4.32) | 4.96 (±4.32) | -1.83 |

Preliminary data for the licenced premises with the largest SD (the student bar) was used to calculate the sample size using the Rstudio software ‘R Stats Package’ and the function power.t.test^[[2]](#endnote-2)^: this provided the most conservative calculation of sample size. Cohen’s d was calculated using the following formula:

$$\boldsymbol{x}\boldsymbol{1-x}\boldsymbol{2}$$

**D =**

$$\sqrt{{\boldsymbol{SD}\boldsymbol{1}}^{\boldsymbol{2}}\boldsymbol{+}{\boldsymbol{SD}\boldsymbol{2}}^{\boldsymbol{2}}}$$

**2**

In line with convention and to make the sample more achievable, the pilot trial was powered at 80% as this elicited a smaller sample size than if it were powered at 90%. The level of statistical significance was set at the 5% level to conform with convention. The SD was set at 1 as this is the default in the R Stats software package.

The figures that were inputted into Rstudio were alpha = 0.05, beta (power) = 0.8, delta (Cohen’s d) = 0.3977, SD = 1. The sample size for a two-sided paired t-test was calculated as 52. Because the study used a cross-over design, this meant that each of the 52 participants would be required to participate in both arms of the trial (two study sessions). The sample size was not inflated to account for potential attrition, so participants who did not provide complete datasets from two study sessions were replaced and their data were destroyed.

1. Office for National Statistics. Adult drinking habits in Great Britain: 2014. 2016. <https://www.ons.gov.uk/peoplepopulationandcommunity/healthandsocialcare/drugusealcoholandsmoking/bulletins/opinionsandlifestylesurveyadultdrinkinghabitsingreatbritain/2014> Accessed: 18 Nov 2016 [↑](#endnote-ref-1)
2. R Core Team. R: A language and environment for statistical computing. 2013. Vienna: R Foundation for Statistical Computing. [↑](#endnote-ref-2)
