# Supplementary material for: The effect of alcohol strength on alcohol consumption: findings from a randomised controlled cross-over pilot trial
Source: Pilot Feasibility Stud. 2021 Jan 30;7:37. doi: 10.1186/s40814-021-00777-4 (PMC7846989; doi:10.1186/s40814-021-00777-4)
Supplement: Supplementary file 2 — Additional file 2. Pilot trial data by recruitment site/gender/student vs non-student. Table of pilot trial data (mean, SD, 95% CIs) by recruitment site/gender/student vs non-student [file 40814_2021_777_MOESM2_ESM.docx]

## Pilot trial data by recruitment site/gender/student vs non-student

|  | **Mean (reduced-strength lager), SD, 95% CI** | **Mean (regular-strength lager), SD, 95% CI** | **Mean difference (mean reduced-strength lager minus mean regular-strength lager), SD, 95% CI** |
| --- | --- | --- | --- |
| **Alcohol consumption**  **(UK units)**  Overall (n=36)  Venue One (n=10)  Venue Two (n=10)  Venue Three (n=10)  Venue Four (n=6)  Female (n=4)  Male (n=32)  Student (n=15)  Non-student (n=21) | 8.28 (SD = 4.17)  (6.87 to 9.69)  8.20 (SD = 3.58)  (5.64 to 10.76)  11.60 (SD = 4.30)  (8.52 to 14.68)  6.80 (SD = 3.01)  (4.65 to 8.95)  5.33 (SD = 1.43)  (1.66 to 9.01)  5.50 (SD = 1.91)  (2.45 to 8.55)  8.63 (SD = 4.26)  (7.09 to 10.16)  6.80 (SD = 3.53)  (4.85 to 8.75)  9.33 (SD = 4.35)  (7.35 to 11.31) | 12.04 (SD = 5.33)  (10.24 to 13.84)  12.51 (SD = 5.26)  (8.75 to 16.27)  14.89 (SD = 5.64)  (10.86 to 18.92)  11.90 (SD = 3.69)  (9.26 to 14.54)  6.75 (SD = 1.67)  (2.45 to 11.05)  7.43 (SD = 1.35)  (5.28 to 9.57)  12.62 (SD = 5.36)  (10.69 to 14.55)  10.45 (SD = 4.58)  (7.92 to 12.99)  13.18 (SD = 5.63)  (10.61 to 15.74) | -3.76 (SD = 3.69)  (-5.01 to -2.52)  -4.31 (SD = 3.52)  (-6.83 to -1.79)  -3.29 (SD = 2.60)  (-5.15 to -1.43)  -5.10 (SD = 4.31)  (-8.18 to -2.02)  -1.42 (SD = 4.01)  (-5.62 to 2.79)  -1.93 (SD = 1.67)  (-4.58 to 0.73)  -3.99 (SD = 3.82)  (-5.37 to -2.62)  -3.65 (SD = 4.60)  (-6.20 to -1.11)  -3.84 (SD = 3.00)  (-5.21 to -2.48) |
| **Alcohol consumption**  **(grams)**  Overall (n=36)  Venue One (n=10)  Venue Two (n=10)  Venue Three (n=10)  Venue Four (n=6)  Female (n=4)  Male (n=32)  Student (n=15)  Non-student (n=21) | 65.78 (SD = 33.51)  (54.44 to 77.12)  65.60 (SD = 28.67)  (45.09 to 86.11)  92.80 (SD = 34.40)  (68.19 to 117.41)  52.80 (SD = 23.91)  (35.70 to 69.90)  42.67 (SD = 28.02)  (13.26 to 72.07)  44.00 (SD = 15.32)  (19.62 to 68.38)  68.50 (SD = 34.30)  (56.13 to 80.87)  53.33 (SD = 28.15)  (37.74 to 68.92)  74.67 (SD = 34.81)  (58.82 to 90.51) | 96.34 (SD = 42.61)  (81.92 to 110.75)  100.08 (SD = 42.04)  (70.00 to 130.16)  119.14 (SD = 45.16)  (86.84 to 151.44)  95.20 (SD = 29.48)  (74.10 to 116.28)  54.00 (SD = 32.76)  (19.62 to 88.38)  59.40 (SD = 10.80)  (42.21 to 76.59)  100.95 (SD = 42.91)  (85.48 to 116.42)  83.62 (SD = 36.59)  (63.36 to 103.88)  105.42 (SD = 45.69)  (84.90 to 125.94) | -30.56 (SD = 29.83)  (-40.65 to -20.46)  -34.48 (SD = 28.20)  (-54.65 to -14.31)  -26.34 (SD = 20.82)  (-41.23 to -11.45)  -42.39 (SD = 34.96)  (-67.40 to -17.38)  -11.33 (SD = 32.07)  (-44.99 to 22.33)  -15.40 (SD = 13.36)  (-36.66 to 5.86)  -32.45 (SD = 30.89)  (-43.59 to -21.32)  -30.29 (SD = 37.48)  (-51.04 to -9.53)  -30.75 (SD = 23.96)  (-41.66 to -19.85) |
| **Pints consumed**  Overall (n=36)  Venue One (n=10)  Venue Two (n=10)  Venue Three (n=10)  Venue Four (n=6)  Female (n=4)  Male (n=32)  Student (n=15)  Non-student (n=21) | 4.14 (SD = 2.09)  (3.43 to 4.84)  4.10 (SD = 1.79)  (2.82 to 5.38)  5.80 (SD = 2.15)  (4.26 to 7.34)  3.4 (SD = 1.51)  (2.32 to 4.48)  2.67 (SD = 1.75)  (0.83 to 4.50)  2.75 (SD = 0.96)  (1.23 to 4.27)  4.31 (SD = 2.13)  (3.54 to 5.08)  3.40 (SD = 1.76)  (2.42 to 4.38)  4.67 (SD = 2.18)  (3.38 to 5.66) | 4.45 (SD = 1.96)  (3.79 to 5.12)  4.63 (SD = 1.94)  (3.24 to 6.02)  5.50 (SD = 2.07)  (4.02 to 6.98)  4.4 (SD = 1.35)  (3.43 to 5.37)  2.50 (SD = 1.52)  (0.91 to 4.09)  2.75 (SD = 0.50)  (1.95 to 3.55)  4.67 (SD = 1.97)  (3.95 to 5.38)  3.87 (SD = 1.68)  (2.93 to 4.80)  4.87 (SD = 2.07)  (3.93 to 5.82) | -0.31 (SD = 1.51)  (-0.82 to 0.20)  -0.53 (SD = 1.34)  (-1.49 to 0.43)  0.30 (SD = 0.95)  (-0.38 to 0.98)  -1.00 (SD = 1.83)  (-2.31 to 0.31)  0.17 (SD = 1.72)  (-1.64 to 1.97)  0.00 (SD = 0.82)  (-1.30 to 1.30)  -0.35 (SD = 1.58)  (-0.92 to 0.22)  -0.47 (SD = 1.92)  (-1.53 to 0.60)  -0.20 (SD = 1.17)  (-0.74 to 0.33) |
| **Study session duration**  **(hh:mm)**  Overall (n=36)  Venue One (n=10)  Venue Two (n=10)  Venue Three (n=10)  Venue Four (n=6)  Female (n=2)  Male (n=24)  Student (n=15)  Non-student (n=11) | 2:33 (SD = 0:51)  (2:12 to 2:53)  N/A  3:07 (SD = 1:05)  (2:20 to 3:54)  2:10 (SD = 0:16)  (1:58 to 2:22)  2:13 (SD = 0:34)  (1:37 to 2:49)  2:02 (SD = 0:10)  (0:27 to 3:37)  2:35 (SD = 0:52)  (2:13 to 2:57)  2:15 (SD = 0:27)  (1:59 to 2:30)  2:57 (SD = 1:06)  (2:12 to 3:42) | 2:39 (SD = 0:52)  (2:18 to 3:00)  N/A  3:01 (SD = 1:14)  (2:08 to 3:54)  2:27 (SD = 0:09)  (2:20 to 2:34)  2:24 (SD = 0:44)  (1:37 to 3:11)  2:37 (SD = 0:03)  (2:05 to 3:09)  2:40 (SD = 0:54)  (2:17 to 3:02)  2:26 (SD = 0.27)  (2:11 to 2:42)  2:57 (SD = 1:11)  (2:09 to 3:45) | -0:06 (SD = 0:41)  (-0:23 to 0:10)  N/A  0:06 (SD = 0:43)  (-0:25 to 0:37)  -0:17 (SD = 0:17)  (-0:29 to -0:04)  -0:10 (SD = 1:04)  (-1:18 to 0:56)  -0:35 (SD = 0:07)  (-1:38 to 0:28)  -0:04 (SD = 0:42)  (-0:22 to 0:13)  -0:11 (SD = 0:45)  (-0:36 to 0:13)  0:00 (SD = 0:37)  (-0:25 to 0:25) |
| **Pleasantness of taste**  Overall (n=36)  Venue One (n=10)  Venue Two (n=10)  Venue Three (n=10)  Venue Four (n=6)  Female (n=4)  Male (n=32)  Student (n=15)  Non-student (n=21) | 4.86 (SD = 2.73)  (3.94 to 5.79)  4.97 (SD = 2.41)  (3.25 to 6.69)  4.17 (SD = 2.32)  (2.51 to 5.83)  5.1 (SD = 3.48)  (2.61 to 7.59)  5.43 (SD = 3.00)  (2.32 to 8.55)  4.50 (SD = 2.30)  (0.85 to 8.15)  4.91 (SD = 2.81)  (3.89 to 5.92)  4.91 (SD = 3.33)  (3.07 to 6.76)  4.82 (SD = 2.30)  (3.78 to 5.87) | 5.81 (SD = 2.13)  (5.09 to 6.53)  5.38 (SD = 2.00)  (3.98 to 6.78)  4.86 (SD = 2.63)  (2.98 to 6.74)  6.73 (SD = 1.74)  (5.49 to 7.97)  6.57 (SD = 1.56)  (4.93 to 8.21)  7.40 (SD = 1.07)  (5.69 to 9.11)  5.61 (SD = 2.16)  (4.83 to 6.39)  6.49 (SD = 1.76)  (5.51 to 7.46)  5.32 (SD = 2.28)  (4.29 to 6.36) | -0.95 (SD = 3.43)  (-2.11 to 0.21)  -0.41 (SD = 3.36)  (-2.81 to 1.99)  -0.69 (SD = 3.32)  (-3.06 to 1.68)  -1.63 (SD = 4.19)  (-4.63 to 1.37)  -1.13 (SD = 2.97)  (-4.25 to 1.99)  -2.90 (SD = 3.03)  (-7.72 to 1.92)  -0.70 (SD = 3.44)  (-1.94 to 0.54)  -1.57 (SD = 3.60)  (-3.57 to 0.42)  -0.50 (SD = 3.32)  (-2.01 to 1.01) |
| **Enjoyment**  Overall (n=36)  Venue One (n=10)  Venue Two (n=10)  Venue Three (n=10)  Venue Four (n=6)  Female (n=4)  Male (n=32)  Student (n=15)  Non-student (n=21) | 4.79 (SD = 2.79)  (3.53 to 5.89)  4.99 (SD = 2.58)  (3.15 to 6.83)  4.40 (SD = 2.66)  (2.49 to 6.31)  5.03 (SD = 3.53)  (2.51 to 7.55)  4.70 (SD = 2.65)  (1.92 to 7.48)  5.50 (SD = 3.01)  (0.71 to 10.29)  4.70 (SD = 2.80)  (3.69 to 5.71)  4.80 (SD = 3.22)  (3.02 to 6.58)  4.78 (SD = 2.53)  (3.63 to 5.94) | 6.23 (SD = 2.21)  (5.40 to 7.27)  5.96 (SD = 1.98)  (4.54 to 7.38)  5.18 (SD = 2.78)  (3.19 to 7.17)  6.86 (SD = 2.03)  (5.41 to 8.31)  7.38 (SD = 1.07)  (6.26 to 8.50)  7.05 (SD = 1.25)  (5.06 to 9.04)  6.13 (SD = 2.30)  (5.30 to 6.96)  6.73 (SD = 2.03)  (5.61 to 7.86)  5.87 (SD = 2.32)  (4.82 to 6.93) | -1.44 (SD = 3.54)  (-2.64 to -0.24)  -0.97 (SD = 3.21)  (-3.27 to 1.33)  -0.78 (SD = 3.53)  (-3.31 to 1.75)  -1.83 (SD = 4.69)  (-5.18 to 1.52)  -2.68 (SD = 1.97)  (-4.75 to -0.61)  -1.55 (SD = 3.89)  (-7.74 to 4.64)  -1.43 (SD = 3.57)  (-2.71 to -0.14)  -1.93 (SD = 3.90)  (-4.09 to 0.23)  -1.09 (SD = 3.32)  (-2.60 to 0.42) |
| **Perceived intoxication**  Overall (n=36)  Venue One (n=10)  Venue Two (n=10)  Venue Three (n=10)  Venue Four (n=6)  Female (n=4)  Male (n=32)  Student (n=15)  Non-student (n=21) | 4.09 (SD = 1.91)  (3.44 to 4.73)  4.90 (SD = 1.83)  (3.59 to 6.21)  4.03 (SD = 1.56)  (2.91 to 5.15)  3.35 (SD = 1.72)  (2.12 to 4.58)  4.05 (SD = 2.70)  (1.22 to 6.88)  4.38 (SD = 0.89)  (2.96 to 5.79)  4.05 (SD = 2.00)  (3.33 to 4.77)  3.85 (SD = 2.16)  (2.66 to 5.05)  4.25 (SD = 1.74)  (3.46 to 5.04) | 5.09 (SD = 1.97)  (4.42 to 5.76)  6.00 (SD = 1.17)  (5.16 to 6.84)  4.39 (SD = 2.01)  (2.95 to 5.83)  5.02 (SD = 1.77)  (3.76 to 6.28)  4.85 (SD = 3.02)  (1.68 to 8.02)  5.65 (SD = 2.05)  (2.39 to 8.91)  5.02 (SD = 1.98)  (4.30 to 5.73)  4.93 (SD = 2.26)  (3.68 to 6.18)  5.20 (SD = 1.79)  (4.39 to 6.02) | -1.00 (SD = 1.79)  (-1.61 to -0.40)  -1.10 (SD = 1.70)  (-2.32 to 0.12)  -0.36 (SD = 1.36)  (-1.33 to 0.61)  -1.67 (SD = 1.74)  (-2.91 to -0.43)  -0.80 (SD = 2.61)  (-3.53 to 1.93)  -1.28 (SD = 2.43)  (-5.13 to 2.58)  -0.97 (SD = 1.75)  (-1.60 to -0.34)  -1.08 (SD = 2.16)  (-2.28 to 0.12)  -0.95 (SD = 1.53)  (-1.65 to -0.25) |
